# Modular pathway engineering for the microbial production of branched-chain fatty alcohols

**DOI:** 10.1186/s13068-017-0936-4

**Published:** 2017-10-27

**Authors:** Wen Jiang, James B. Qiao, Gayle J. Bentley, Di Liu, Fuzhong Zhang

**Affiliations:** 1 Department of Energy, Environmental and Chemical Engineering, Washington University in St. Louis, 1 Brookings Drive, Campus Box 1180, Saint Louis, MO 63130 USA; 2 Division of Biological & Biomedical Sciences, Washington University in St. Louis, Saint Louis, MO 63130 USA; 3 Institute of Materials Science & Engineering, Washington University in St. Louis, Saint Louis, MO 63130 USA; 4 Present Address: National Bioenergy Center, National Renewable Energy Laboratory, Golden, CO 80401 USA

**Keywords:** Branched long-chain fatty alcohols, Branched-chain fatty acids, Advanced biofuels, Modular pathway engineering

## Abstract

The intrinsic structural properties of branched long-chain fatty alcohols (BLFLs) in the range of C12 to C18 make them more suitable as diesel fuel replacements and for other industrial applications than their straight-chain counterparts. While microbial production of straight long-chain fatty alcohols has been achieved, biosynthesis of BLFLs has never been reported. In this work, we engineered four different biosynthetic pathways in *Escherichia coli* to produce BLFLs. We then employed a modular engineering approach to optimize the supply of α-keto acid precursors and produced either odd-chain or even-chain BLFLs with high selectivity, reaching 70 and 75% of total fatty alcohols, respectively. The acyl-ACP and alcohol-producing modules were also extensively optimized to balance enzyme expression level and ratio, resulting in a 6.5-fold improvement in BLFL titers. The best performing strain overexpressed 14 genes from 6 engineered operons and produced 350 mg/L of BLFLs in fed-batch fermenter. The modular engineering strategy successfully facilitated microbial production of BLFLs and allowed us to quickly optimize new BLFL pathway with high titers and product specificity. More generally, this work provides pathways and knowledge for the production of BLFLs and BLFL-related, industry-relevant chemicals in high titers and yields.

**Electronic supplementary material:**

The online version of this article (doi:10.1186/s13068-017-0936-4) contains supplementary material, which is available to authorized users.

## Background

Finite energy resources and increased environmental concerns demand the development of sustainable and renewable approaches to the production of fuels, chemicals, and materials. Engineering microbial metabolic pathways to synthesize desired products is an attractive approach. With recent advances in genetic techniques and metabolic engineering methodologies, various engineered microbes have been developed to produce an array of chemicals derived from inexpensive and renewable substrates [1, 2]. Target molecules are typically designed to replace or mimic those obtained from petroleum or other non-renewable sources [3, 4]. Among these chemicals, long-chain fatty alcohols (LCFLs) in the range of C12–C18 have numerous applications as fuels, emollients, plasticizers, thickeners, and detergents [5–7]. At the industrial scale, LCFLs are currently produced either through hydrogenation of pretreated natural fats and oils (oleochemicals) or hydroformylation of petrochemicals (e.g. crude oil, natural gas) [8–10]. Because both processes require harsh reaction conditions and release harmful byproducts to the environment [11], microbial production of fatty alcohols from renewable sugars is a promising alternative.

Long-chain fatty alcohols have been recently biosynthesized from engineered microbes through fatty acid biosynthetic pathway [5, 10–15]. The majority of previously biosynthesized LCFLs contain straight aliphatic chains. Straight LCFLs have relatively high freezing points and viscosities compared to their branched-chain isomers, thus limiting their low-temperature operability—an essential feature for industrial emollients, lubricants, detergents, and most especially, diesel fuels [16, 17]. For example, the most abundant biosynthetically produced LCFL, straight-chain palmityl alcohol (C16, often called cetyl alcohol), melts at 49.3 °C and exists as a solid at room temperature, making it difficult to use as a lubricant or an aircraft hydraulic fluid [18]. In comparison, the branched isomer, isocetyl alcohol, is a clear liquid at room temperature (melting point − 30 °C) with significantly enhanced fluidity and low-temperature operability, and thus is used in a number of detergent formulations that require low-temperature operability [19]. Introducing degrees of unsaturation has been used as an alternative method to improve the fluidity of straight-chain molecules [20]. However, these molecules are vulnerable to oxidation and color instability, both of which do not occur in BLFLs, even at high temperatures and pressures [17]. BLFLs additionally are ranked low in eye and skin irritation, allowing their use in cosmetics and fragrances [16]. Given the clear breadth BLFL applications, chemical engineers have developed methods to synthesize naturally scarce BLFLs [21–23]. BLFLs are currently produced from straight LCFLs using a multi-step, catalytic process that requires high temperature (180–300 °C), strong acids/bases, and multiple operation unites, and the conversion process is unpredictable [16]. Recently, branched-short-chain alcohols in the range of (C3–C7) were produced by engineered microbes [24, 25] as gasoline replacements. However, due to their short chain-length and high volatility, branched-short-chain alcohols are not suitable for the above-mentioned applications. Given BLFLs’ lack of natural abundance, their complex synthesis process, and the demand for their distinctive properties, it is imperative to develop biosynthetic approaches to produce BLFLs from renewable feedstock.

Since straight LCFLs have been biologically or chemically derived from free fatty acid precursors [26], BLFLs theoretically can be derived from branched-chain fatty acids (BCFAs). BCFAs have been recently biosynthesized in engineered *E. coli* by overexpressing a heterologous branched-chain α-keto acid dehydrogenase complex (BKD), a branched-chain-acyl-CoA-specific β-ketoacyl-acyl-carrier protein (FabH) and a thioesterase (TesA) [27, 28]. This system ultimately produced BCFA in high percentages from glucose (181 mg/L and 80% BCFA) by engineering a protein lipoylation pathway and an α-keto acid biosynthetic pathway. However, highly efficient conversion of BCFAs to BLFLs has not been explored. It was not known whether the branched-chain intermediates, such as branched long-chain acyl-ACPs, BCFAs, and branched long-chain acyl-CoAs, are compatible with alcohol-conversion enzymes, even though some enzymes have activities towards branched short-chain-ACP or CoA intermediates [29, 30]. Furthermore, because the BLFL pathway involves in multiple untested reaction, balancing activities of each sub-pathway to prevent accumulation of branched-chain intermediate is necessary to obtain high titer and yield [31–34].

**Fig. 1:**
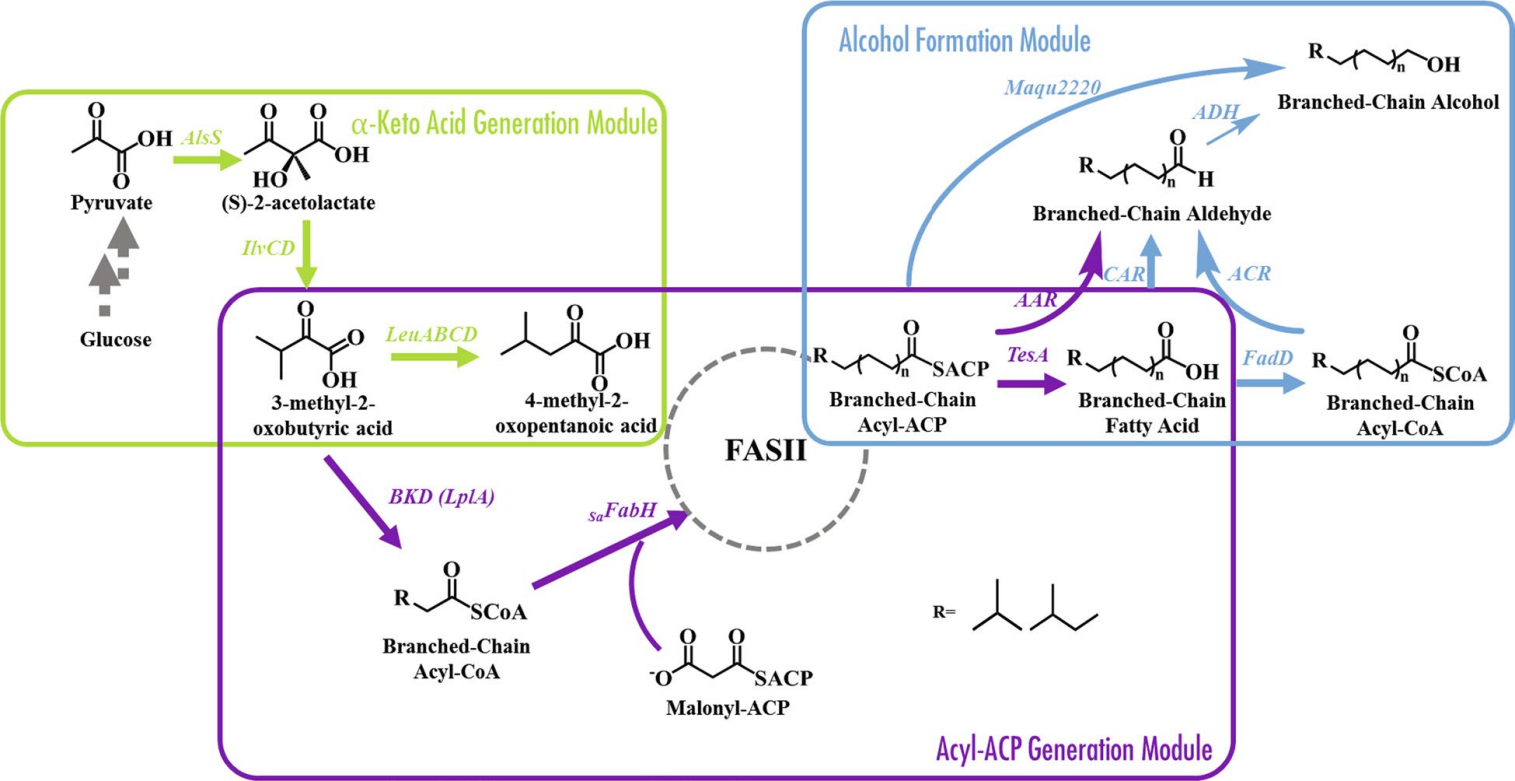
Biosynthetic pathways for BLFL production. α-Keto acid generation module converts pyruvate to α-keto acid: 3-methyl-2-oxobutyric acid or 4-methyl-2-oxopentanoic acid. Acyl-ACP generation module incorporates α-keto acid to the fatty acid biosynthetic pathway. Alcohol formation module converts branched-chain acyl-ACPs to BLFLs

To achieve these goals, we constructed and tested four metabolic pathways that convert branched-chain acyl-ACPs to BLFLs. To optimize productivity, the entire BLFL pathway was divided into three modules (Fig. 1): an α-keto acid synthesis module that converts glucose to α-keto acids, an acyl-ACP generation module that converts α-keto acids to branched-chain acyl-ACPs, and an alcohol formation module that converts branched-chain acyl-ACPs to final branched-chain products. To begin, we experimentally determined the most efficient enzyme combinations to convert branched-chain acyl-ACPs to BLFLs using an engineered branched-chain-acyl-ACP-producing basal strain (BC33). This initial pathway identification allowed us to ascertain whether BLFL production was even feasible, given the available enzymes. Next, we optimized each module separately and later the entire pathway collectively to produce even-chain-iso and odd-chain-iso fatty alcohols from glucose. Our modular approach enabled us to isolate each module for testing and optimization, without confounding the system by simultaneously engineering the other modules.

## Methods

### Materials and media

Phusion DNA polymerase was purchased from New England Biolabs (Beverly, MA, USA). Restriction enzymes, T4 ligase, gel purification kits, and plasmid miniprep kits were purchased from Thermo Fisher Scientific (Waltham, Massachusetts, USA). All primers were synthesized by Integrated DNA Technologies (Coralville, IA, USA). BCFA standards (Bacterial Acid Methyl Ester Mix), SCFA standards (GLC-20 and GLC-30), and all the other reagents were purchased from Sigma Aldrich (St. Louis, MO, USA).

Minimal medium (M9 medium supplemented with 75 mM MOPS at pH 7.4, 2 mM MgSO_4_, 1 mg/L thiamine, 50 μg/mL lipoic acid, 10 μM FeSO_4_, 0.1 mM CaCl_2_, and micronutrients, including 3 μM (NH_4_)_6_Mo_7_O_24_, 0.4 mM boric acid, 30 μM CoCl_2_, 15 μM CuSO_4_, 80 μM MnCl_2_, and 10 μM ZnSO_4_) containing 2% glucose and 0.5% yeast extract as carbon sources was used for cell growth and fatty acid production.

### Plasmids and strains

Plasmids and oligonucleotides used in this study are listed in Table 1. Genes encoding *S. elongatus* AAR (*aar*), *M. marinum* CAR (*car*), and *B. subtilis* Sfp (*sfp*) were codon-optimized for *E. coli* expression and synthesized by Integrated DNA Technologies (Coralville, IA, USA). Genes encoding ACR (*maqu2507*) and Maqu2220 (*maqu2220*) were amplified by PCR from templates provided by Dr. Brett M. Barney (University of Minnesota, MN) and Dr. Jay D. Keasling (Joint BioEnergy Institute, CA). To create all plasmids (Table 1), the corresponding genes were assembled into the backbones of BioBrick plasmids [35], using either restriction sites or Golden-Gate DNA assembly method [36].

**Table 1:** Plasmids used in this research

Plasmids	Replication ori	Overexpressed operon	Resistance	References
pSa-P_ecfabH_-_*Sa*_ *fabH*	SC101	P_ecfabH_-_Sa_ *fabH* (*S. aureus*)	Amp^R^	[28]
pB5k-*aar*-*lplA*	pBBR1	P_lacUV5_-*aar* (*S. elongatus*)-*lplA* (*E. coli*)	Kan^R^	This study
pB5k-*sfp*-*car*	pBBR1	P_lacUV5_-*sfp*(*B. subtilis*)-*car* (*M. marinum*)	Kan^R^	This study
pE8c-*tesA*-*lplA*	ColE1	P_BAD_-*tesA* (*E. coli*)-*lplA* (*E. coli*)	Cm^R^	This study
pB5k-*maqu2507*-*lplA*	pBBR1	P_lacUV5_-*maqu2507* (*M. aquaeolei* VT8)-*lplA* (*E. coli*)	Kan^R^	This study
pE5c-*tesA*-*fadD*	ColE1	P_lacUV5_-*tesA* (*E. coli*)-*fadD* (*E. coli*)	Cm^R^	[48]
pB5k-*maqu2220*	pBBR1	P_lacUV5_-*maqu2220* (*M. aquaeolei* VT8)	Kan^R^	[48]
pB5k-*maqu2220*-*lplA*	pBBR1	P_lacUV5_-*maqu2220* (*M. aquaeolei* VT8)-*lplA* (*E. coli*)	Kan^R^	This study
pB5k-*adhA*	pBBR1	P_lacUV5_-*adhA* (*L. lactis*)	Kan^R^	This study
pB5k-*yqhD*	pBBR1	P_lacUV5_-*yqhD* (*E. coli*)	Kan^R^	This study
pB5k-*yjgB*	pBBR1	P_lacUV5_-*yjgB* (*E. coli*)	Kan^R^	This study
pA8c-*aar*-*lplA*	pA15a	P_BAD_-*aar* (*S. elongatus*)-*lplA* (*E. coli*)	Cm^R^	This study
pB2k-*alsS*-*ilvCD*-*yjgB*	pBBR1	P_tet_-*alsS* (*B. subtilis*)-*ilvCD* (*E. coli*)-*yjgB*(*E. coli*)	Kan^R^	This study
pE2c-*aar*-*lplA*-*yjgB*	ColE1	P_tet_-*aar* (*S. elongatus*)-*lplA* (*E. coli*)-*yjgB* (*E. coli*)	Cm^R^	This study
pA6k-*alsS*-*ilvCD*	pA15a	P_LlacO_-*alsS* (*B. subtilis*)-*ilvCD* (*E. coli*)	Kan^R^	[43]
pB2k-*alsS*-*ilvCD*	pBBR1	P_tet_-*alsS* (*B. subtilis*)-*ilvCD* (*E. coli*)	Kan^R^	This study
pB5c-*aar*-*lplA*-*yjgB*	pBBR1	P_lacUV5_-*aar* (*S. elongatus*)-*lplA* (*E. coli*)-*yjgB* (*E. coli*)	Cm^R^	This study
pB5c-*maqu2220*-*lplA*	pBBR1	P_lacUV5_-*maqu2220* (*M. aquaeolei* VT8)-*lplA* (*E. coli*)	Cm^R^	This study
pA8c-*leuA* ^*mut*^ *BCD*	pA15a	P_BAD_-*leuA* ^*mut*^ *BCD* (*E. coli*)	Cm^R^	This study
pE8c-*leuA* ^*mut*^ *BCD*	ColE1	P_BAD_-*leuA* ^*mut*^ *BCD* (*E. coli*)	Cm^R^	This study
pB5k-*maqu2220*-*leuA* ^*mut*^ *BCD*	pBBR1	P_lacUV5_-*maqu2220* (*M. aquaeolei* VT8)-*leuA* ^*mut*^ *BCD* (*E. coli*)	Kan^R^	This study
pA8c-*lplA*	pA15a	P_BAD_-*lplA* (*E. coli*)	Cm^R^	This study
pE8c-*adhA*	ColE1	P_BAD_-*adhA* (*L. lactis*)	Cm^R^	This study
pE8c-*yqhD*	ColE1	P_BAD_-*yqhD* (*E. coli*)	Cm^R^	This study
pE8c-*yjgB*	ColE1	P_BAD_-*yjgB* (*E. coli*)	Cm^R^	This study
pE2s-*alsS*-*ilvCD*	ColE1	P_tet_-*alsS* (*B. subtilis*)-*ilvCD* (*E. coli*)	Spec^R^	This study

*Escherichia coli* DH10B was used for cloning purposes. *E. coli* strain CL111 [37] (a gift from Dr. Cronan’s Lab, University of Illinois at Urbana-Champaign) was used for production purposes. Strain BC30 was created by integrating the *bkd* operon (*lpdV, bkdAA, bkdAB,* and *bkdB*) at the *fadE* locus in the genome under the control of a P_LacUV5_ promoter, using a previously described technique [38]. Plasmid pSa-P_ecfabH_-_Sa_fabH was transformed into BC30 strain, and the _*Se*_ *fabH* was knocked out by P1 transduction, creating strain BC33 [37]. Strains BO33A-J were created by transforming the corresponding plasmids (Table 2) into BC33 competent cells, respectively. Strain BC43 was created by switching native *leuABCD* promoter P_leuLp_ and P_leuLp2_ to P_lacUV5_ promoter, using a previously described CRISPR-Cas9 gene replacement method [39]. Similarly, to create strain BC63, the *fadR*-*lplA* operon under the control of a P_LacUV5_ promoter was integrated to the *ldhA* locus of BC43 strain by the same CRISPR-Cas9 gene replacement method. Strains BO63V and BO63L were created by transforming the corresponding plasmids into strain BC63.

**Table 2:** Strains used in this research

Strains	Relevant genotype	References
Parental strains		
CL111	UB1005, attHK022::(*plsX’fabH*; *aadA*) *fabH*::kan	[37]
CL111(Δ*kan*)	UB1005, attHK022::(*plsX’fabH*; *aadA*), Δkan	[28]
BC30	CL111(Δ*kan*) *fadE*:: _*bs*_ *lpdV*-_*bs*_ *bkdAA*-_*bs*_ *bkdAB*-_*bs*_ *bkdB*	This study
BC33	CL111(Δ*kan*) (*plsX*’*fabH*; *aadA*)::Tet^A^ *fadE*:: _*bs*_ *lpdV*-_*bs*_ *bkdAA*-_*bs*_ *bkdAB*-_*bs*_ *bkdB* pSa-P_ecfabH_-_Sa_ *fabH*	This study
BC43	BC33 *leuO*-*leuL*-*leuA::P*_*lacUV5*_-*leuA* ^*mut*^	This study
BC63	BC43 *ldhA*::*fadR*-*lplA*	This study
Alcohol-producing strains		
BO33A	BC33 pB5k-*aar*-*lplA*	This study
BO33B	BC33 pB5k-*sfp*-*car*, pE8c-*tesA*-*lplA*	This study
BO33C	BC33 pB5k-*maqu2507*-*lplA*, pE5c-*tesA*-*fadD*	This study
BO33D	BC33 pB5k-*maqu2220*-*lplA*	This study
BO33E1	BC33 pA8c-*aar*-*lplA*, pB5k-*adhA*	This study
BO33E2	BC33 pA8c-*aar*-*lplA*, pB5k-*yqhD*	This study
BO33E3	BC33 pA8c-*aar*-*lplA*, pB5k-*yjgB*	This study
BO33E4	BC33 pE2c-*aar*-*lplA*-*yjgB*	This study
BO33F	BC33 pA8c-*aar*-*lplA*, pB2k-*alsS*-*ilvCD*-*yjgB*	This study
BO33F1	BC33, pE2c-*aar*-*lplA*-*yjgB*, pA6k-*alsS*-*ilvCD*	This study
BO33F2	BC33, pE2c-*aar*-*lplA*-*yjgB*, pB2k-*alsS*-*ilvCD*	This study
BO33F3	BC33, pB5c-*aar*-*lplA*-*yjgB*, pA6k-*alsS*-*ilvCD*	This study
BO33G1	BC33, pA8c-*leuA* ^*mut*^ *BCD*, pB5k-*aar*-*lplA*	This study
BO33G2	BC33, pE8c-*leuA* ^*mut*^ *BCD*, pB5k-*aar*-*lplA*	This study
BO33H1	BC33 pB5k-*maqu2220*-*lplA*, pE8c-*adhA*	This study
BO33H2	BC33 pB5k-*maqu2220*-*lplA*, pE8c-*yqhD*	This study
BO33H3	BC33 pB5k-*maqu2220*-*lplA*, pE8c-*yjgB*	This study
BO33I1	BC33, pA6k-*alsS*-*ilvCD*, pB5c-*maqu2220*-*lplA*	This study
BO33I2	BC33, pE2k-*alsS*-*ilvCD*, pB5c-*maqu2220*-*lplA*	This study
BO33J1	BC33, pA8c-*leuA* ^*mut*^ *BCD*, pB5k-*maqu2220*-*lplA*	This study
BO33J2	BC33, pA8c-*lplA*, pB5k-*maqu2220*-*leuA* ^*mut*^ *BCD*	This study
BO43I	BC43 pB5c-*maqu2220*-*lplA*, pE2s-*alsS*-*ilvCD*	This study
BO63V	BC63 pB5k-*maqu2220*, pE2s-*alsS*-*ilvCD*	This study
BO63L	BC63 pB5k-*maqu2220*, pE2s-*alsS*-*ilvCD*, pA8c-*leuABCD*	This study

### Cell culturing and α-keto acids supplementation

Cells were pre-cultivated in LB medium with proper antibiotics. Overnight cultures were inoculated 2% v/v into M9 minimal medium (described in “Materials and media” section) with corresponding antibiotics for adaptation. Overnight cultures in minimal medium were then used to inoculate 5 mL of the same fresh minimal medium, with an initial OD_600_ of 0.08. When OD_600_ reached 0.8, cells were induced with proper inducers (1 mM isopropyl β-d-1-thiogalactopyranoside (IPTG), 0.4% arabinose and/or 200 nM anhydrotetracycline (aTc), or otherwise specified). For α-keto acid supplementation experiments, one of the α-keto acids (3-methyl-2-oxobutyric acid, 3-methyl-2-oxopentanoic acid, or 4-methyl-2-oxopentanoic acid) was added at OD_600_ = 0.8 to a final concentration of 1 g/L. Cells were harvested 3 days after induction.

### Fermentation

Fed-batch fermentation was carried out using a New Brunswick Bioflo 110 fermenter with a pH meter, a dissolved oxygen electrode, and a temperature electrode. M9 medium (described in “Materials and media” section, 500 mL) was inoculated with an overnight culture of strain BO63L to an initial OD_600_ to 0.08, along with appropriate antibiotics and 0.001% Antifoam 204. The fermentation was initiated with the following settings: Temperature was set to 30 °C, pH was controlled at 7.4 by automatic feeding of 6 N ammonium hydroxide, the airflow rate was kept at 1.5 L/min, and the average stirring rate was maintained at 500 rpm. Gene expression was induced at OD_600_ = 10 by addition of 1 mM IPTG, 0.4% arabinose, and 0.22 μM aTc (final concentration). A glucose stock solution (400 g/L glucose and 12 g/L MgSO_4_) was intermittently pulsed into the bioreactor to re-supply glucose, and a yeast extract solution (20%) was intermittently added into the bioreactor. Broth samples (~ 3 mL) were collected at a series of time points to measure cell density and alcohol titer.

### Quantification of the fatty alcohols

For the quantification of alcohols, 1 mL of cell culture was acidified with 100 μL of concentrated HCl (12 N). Alcohols were extracted twice with 0.5 mL ethyl acetate, and the organic layers were isolated. Next, 200 μL of the organic layer from each sample was transferred to one 2 mL clear glass GC vial (Agilent Technologies, Santa Clara, CA), mixed with 200 μL of *N*,*O*-bis(trimethylsilyl)trifluoroacetamide (BSTFA) with 1% v/v chlorotrimethylsilane, and incubated at 60 °C for 2 h. Alcohol derivatives were quantified using a GC–MS (Hewlett-Packard model 7890 A, Agilent Technologies) equipped with a 30 m DB5-MS column (J&W Scientific) and a mass spectrometer (5975C, Agilent Technologies) or a FID (Agilent Technologies) detector. For each sample, the column was equilibrated at 80 °C, followed by a ramp to 300 °C at 20 °C/min, and was then held at 300 °C for 3 min. Individual alcohol peaks were identified by comparing their retention time to that of a standard (a mixture of 1-tetradecanol, 1-hexadecanol, and 1-octadecanol, prepared and derivatized identically to samples) and by comparing their mass spectra to the National Institute of Standards and Technology (NIST) Mass Spectral Library. Concentrations of each alcohol were determined by comparing the area of each sample peak to a standard curve generated by standards eluted using the same method. Product titer for each strain was measured in biological triplicate (starting from three different colonies) and average values are reported.

## Results

### Engineering alcohol formation modules in BCFA-producing strains

To create the branched-chain-acyl-ACP-producing basal strain BC33, the *E. coli fabH* was first replaced by *Staphylococcus aureus fabH* (_*Sa*_ *fabH*), a modification which was previously demonstrated to enhance branched-chain fatty acid production [28]. The acyl-CoA dehydrogenase gene (*fadE*) was next replaced by *Bacillus subtilus bkd*, functionally inhibiting β-oxidation (Δ*fadE*) and allowing the activation of branched-chain α-keto acids to branched-chain acyl-CoAs that can enter the FASII system (*fadE::bkd*) (Tables 1, 2). Next, three alcohol formation modules were separately constructed in strain BC33 (Table 2), resulting in alcohol-producing strains BO33A-C. Strain BO33A utilizes the *Synechococcus elongatus* acyl-ACP reductase (AAR, encoded by *aar*, Fig. 1) to convert acyl-ACPs to fatty aldehydes [13]. Strain BO33B first generates FFAs via expression of the *E. coli* cytosolic thioesterase (TesA), which are then converted to fatty aldehydes by the *Mycobacterium marinum* carboxylic acid reductase (CAR, encoded by *car*) with *Bacillus subtilis* Sfp (encoded by *sfp*) coexpression [5] (Fig. 1). Strain BO33C also expresses cytosolic TesA to generate FFAs and overexpresses an *E. coli* acyl-CoA synthetase (FadD) to activate FFAs to fatty acyl-CoAs, which are then converted to aldehydes by overexpressing an acyl-CoA reductase from *Marinobacter aquaeolei* VT8 (ACR, encoded by *maqu2507*) [40] (Fig. 1). All strains rely on native *E. coli* alcohol dehydrogenases (ADHs) to reduce aldehydes to alcohols. The native *E. coli* lipoyl ligase (encoded by *lplA*) is also expressed in all strains to improve the lipoylation of 2-oxoacid dehydrogenases including BKD, which requires lipoylation to function [27].

**Fig. 2:**
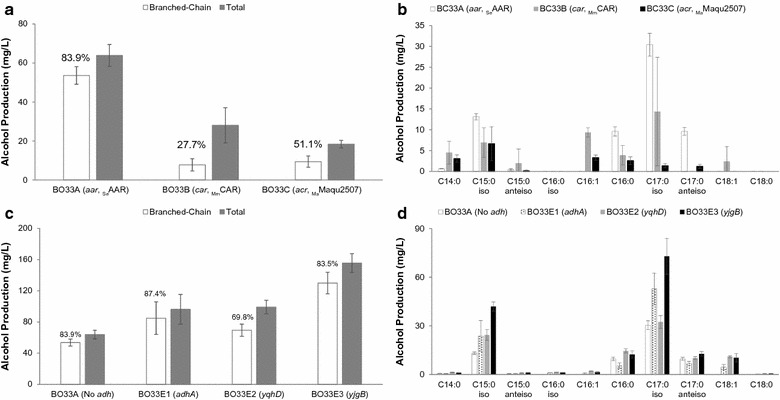
Testing three alcohol formation modules. **a** Overall titer distribution and **b** chain-length of three BLFL-producing strains with different alcohol formation modules, BO33A-C (Table 2). _Se_AAR, _Mm_CAR, and _Ma_Maqu2507 are reductases from *S. elongatus*, *M. marinum*, and *M. aquaeolei* VT8, respectively. **c**, **d** Effect of alcohol dehydrogenase overexpression on BLFL production. Alcohol profiles of strain BO33A (without *adh* overexpression) are compared with those from strain BO33E1 (expressing *adhA* from *L. lactis*), BO33E2 (expressing *yqhD* from *E. coli*), and BO33E3 (expressing *yjgB* from *E. coli*). All cultures were supplemented with 1 g/L 4-methyl-2-oxopentanoic acid. Cells were cultivated and induced as described in “Methods” section

We first sought to test the three alcohol formation modules by assessing their capacities to convert branched-chain acyl-ACPs to BLFLs. Strains BO33A-C were cultivated at previously determined optimal temperatures for each pathway (see “Methods” section) [5, 12, 41] and supplemented with 1 g/L of 4-methyl-2-oxopentanoic acid, the most favorable α-keto acids for BCFA production [28]. Each strain produced some BLFLs, representing the first report of BLFL production in *E. coli.* The CAR pathway (BO33B) and the ACR pathway (BO33C) produced 28 and 18 mg/L BLFLs, respectively. In addition, the chain lengths of the products range from C14 to C18, consistent with the FFA profile of the TesA-overexpressing strain [42]. Meanwhile, the AAR pathway (BO33A) produced the highest BLFL titer among these three strains: 54 mg/L, comprising 84% of the total fatty alcohols (Fig. 2a). The branch and chain-length profile of BLFLs produced in BO33A is consistent with the profile of BCFAs produced by strains with similar genetic backgrounds as previously reported (Fig. 2b) [27], indicating that the AAR pathway has little preference between straight-chain and branched-chain acyl-ACP substrates. Interestingly, the CAR pathway produced twofold more straight-chain fatty alcohols but sixfold less BLFLs than the AAR pathway, indicating that the CAR pathway is primarily straight-chain-specific. While these results serve as a proof-of-principle that BLFL can be produced at high percentages in *E. coli,* the initial low titers indicate that additional engineering efforts are needed to obtain desirable titers and yields of branched-chain products.

All of the above strains relied upon *E. coli* native ADHs to convert fatty aldehydes to alcohols. We next sought to increase BLFL production in strain BO33A by overexpression of ADHs. Three unique ADHs, *adhA* from *Lactococcus lactis* [43], and *yqhD* and *yjgB* [5, 43, 44] from *E. coli*, were overexpressed in strain BO33A, resulting in strains BO33E1, BO33E2, and BO33E3, respectively. When cultivated under the same conditions and with 1 g/L 4-methyl-2-oxopentanoic acid, strains BO33E1 and BO33E2 produced 85 and 69 mg/L BLFLs, respectively (Fig. 2c, d). Strain BO33E3 produced 130 mg/L BLFLs, comprising 83% of total alcohols, a 2.4-fold enhancement over BO33A. Specifically, 73 mg/L of 15-methylhexadecanol and 42 mg/L of 13-methyltetradecanol were produced (Fig. 2c), converting 8% of the supplemented 4-methyl-2-oxopentanoic acid and making odd-chain-iso alcohols the major BC alcohol species. Both BO33E1 and BO33E3 had similar fatty alcohol production profiles to that of strain BO33A, indicating that AdhA and YjgB did not selectively determine chain length or structure. In addition, no aldehyde was detected in BO33E3 culture, indicating the conversion of aldehyde to alcohol is complete.

### Engineering 3-methyl-2-oxobutyric acid generation module to produce even-chain-iso fatty alcohols from glucose

**Fig. 3:**
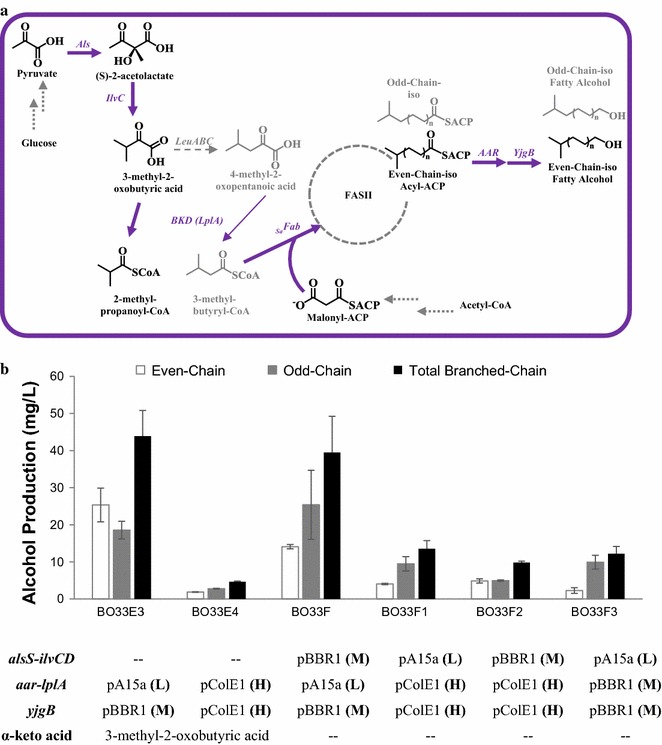
Balancing three modules to produce even-chain-iso fatty alcohols. Plasmids with different copy numbers and promoters are used to optimize the expression level of the involved pathways. **a** Biosynthetic pathways for the production of even-chain-iso fatty alcohols. **b** Titers of even-chain, odd-chain, and total BLFL of engineered strains. Plasmids with different copy numbers and promoters were employed to bear the involved genes in different strains. The copy number of the plasmids are defined as low (L), medium (M), and high (H). For strain BO33E3-4, 1 g/L 3-methyl-2-oxobutyric acid was supplemented

Modular pathway assembly can facilitate step-wise optimizations. With the downstream alcohol formation module optimized, we next sought to improve the production of BLFL from glucose by engineering and optimizing the α-keto acid generation module. In this module, we built upon the downstream-optimized strain BO33E3 to add the capacity for even-chain-iso fatty alcohol production from glucose via the precursor 3-methyl-2-oxobutyric acid (Fig. 3a).

Since the *B. subtilis alsS* and the *E. coli ilvCD* have been used to accumulate intracellular 3-methyl-2-oxobutyric acid, we first cloned them with *yjgB* under the control of a strong P_tet_ promoter in a medium-copy-number BBR1 origin plasmid (Table 1). The resulting plasmid, pB2k-*alsS*-*ilvCD*-*yjgB*, was co-transformed with pA8c-*aar*-*lplA* (*aar* and *lplA* under the control of a P_BAD_ promoter in a pA15a origin plasmid) into strain BC33. Cultivation of the resulting strain BO33F in the absence of α-keto acid supplementation produced 34 mg/L of BLFLs, comprising 44% of the total alcohols (Fig. 3b; Additional file 1: Figure S1). Because the composition of the α-keto acid determines the final chain structure, the final BLFL composition should mirror the cellular pool of α-keto acids. The titer of even-chain-iso BLFLs produced by strain BO33F is 14 mg/L, lower than the 25 mg/L produced by strain BO33E3 when supplemented with 3-methyl-2-oxobutyric acid. However, BO33F produced more odd-chain-iso BLFLs, which are also originated from 3-methyl-2-oxobutyric acid via 4-methyl-2-oxopentanoic acid. Thus the total BLFL production of these two strains is comparable, indicating that the 3-methyl-2-oxobutyric acid generation module was effective, although higher expression level of this module might generate more even-chain-iso products.

### Optimizing 4-methyl-2-oxopentanoic acid generation module to produce odd-chain-iso fatty alcohols

**Fig. 4:**
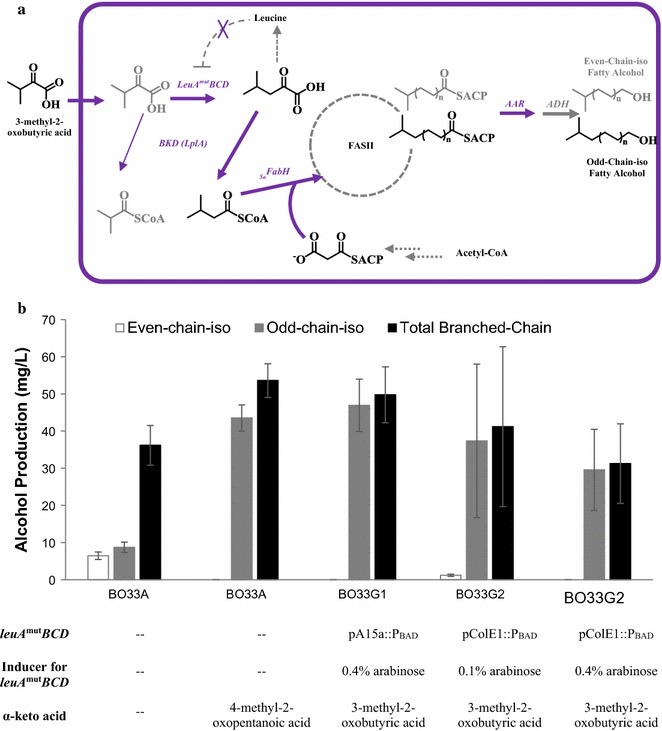
Optimizing the 4-methyl-2-oxopentanoic acid generation module to produce odd-chain-iso fatty alcohols. **a** Biosynthetic pathways for the production of odd-chain-iso fatty alcohols. **b** Titers of engineered odd-chain-iso BLFL-producing strains. Strains BO33G1-2 containing *leuA* ^*mut*^ *BCD* in plasmids with different copy numbers were cultivated as described in “Methods”, and supplemented with 1 g/L 3-methyl-2-oxobutyric acid. Strain BO33G2 was induced with either 0.1 or 0.4% arabinose

In *E. coli*, the *leuABCD* operon converts 3-methyl-2-oxobutyric acid to 4-methyl-2-oxopentanoic acid, which is the precursor for odd-chain-iso BLFL biosynthesis. Previous work demonstrated that the ribosome binding sequence (RBS) of native *leuA* results in weak translation initiation, and *leuA* is heavily negatively auto-regulated by free leucine synthesized from 4-methyl-2-oxopentanoic acid [45, 46] (Fig. 4a). In this study, we engineered a mutant operon *leuA* ^*mut*^ *BCD* that contained a feedback-resistant *leuA* [46, 47] and a strong synthetic RBS (TTTAAGAAGGAGATATACAT). The *leuA* ^mut^ *BCD* was cloned in either a low (pA15a) or a high (pColE1) copy number plasmid to create two engineered strains, BO33G1 and BO33G2, respectively. To test the conversion efficiency of the *leuA* ^*mut*^ *BCD* operon, both strains were supplemented with 3-methyl-2-oxobutyric acid (the substrate for LeuABCD, Fig. 4a) during cultivation. Fully functional *leuA* ^*mut*^ *BCD* would result in odd-chain-iso BLFL compositions in a similar ratio to our best-performing strain (strain BO33A) when supplemented with 4-methyl-2-oxopentanoic acid. Strain BO33G1 produced 50 mg/L total BLFL and 47 mg/L odd-chain-iso fatty alcohol, statistically indistinguishable from the BLFL titer of strain BO33A supplemented with 4-methyl-2-oxopentanoic acid (Fig. 4b; Additional file 1: Figure S2). Strain BO33G1 generated 94% odd-chain-iso fatty alcohol, indicating complete conversion of 3-methyl-2-oxobutyric acid by the modified LeuA^mut^BCD. Conversely, when *leuA* ^mut^ *BCD* was expressed under the high copy number plasmid (strain BO33G2), the BLFL titers decreased (*p* = 0.07) and had large standard variations (Fig. 4b), even when the inducer concentration was reduced. Furthermore, overexpression of *leuA* ^mut^ *BCD* from the high-copy number plasmid also decreased cell growth rate and cell density (data not shown). These results indicate that expression of the *leuA* ^mut^ *BCD* operon at too high a level affects cell growth and BLFL production, thus needs to be avoided.

### Balancing gene expression levels in all three modules for enhanced BLFL production from glucose

With the α-keto acid generation and the alcohol formation modules optimized separately, we next sought to balance the gene expression levels within the completed pathway. We selected the best-performing genetic elements of each individual module to complete the full pathway: the α-keto acid generation module, including *alsS* and *ilvCD*, the acyl-ACP synthesis module, including *aar* and *lplA*, and the alcohol formation module, including *yjgB* (Fig. 3a). To perform this optimization, we altered gene copy numbers to change the expression level of each module. Compatible plasmids with three different copy numbers were used for this purpose: a high copy number (pColE1, 50 copies per cell), a medium copy number (pBBR1, 17–20 copies per cell), and a low copy number (pA15a, 7–10 copies per cell) [35] (Fig. 3b; Additional file 1: Figure S1). Three additional strains BO33E4, BO33F1, and BO33F2 were created (Fig. 3b; Additional file 1: Figure S1). All three strains expressed *aar*, *lplA*, and *yjgB* from the high copy number plasmids under the control of a very tight promoter (P_tet_), but produced less BLFL than strains BO33E3 and BO33F, which expressed *aar*, *lplA*, and *yjgB* from the low or the medium copy number plasmid. The low titer is potentially caused by the insolubility of highly overexpressed AAR [13]. Meanwhile, compared to strain BO33F3 with the low-copy *alsS*-*ilvCD* expression, strain BO33F with the medium-copy *alsS*-*ilvCD* expression improved BLFL production by 3.2-fold. These results suggest that the optimal *aar* expression level was medium to low, and that *alsS*-*ilvCD* required overexpression in a medium to high-copy number plasmid for optimal BLFL production (Fig. 3b; Additional file 1: Figure S1).

### BLFL production from glucose using the acyl-ACP reductase Maqu2220

**Fig. 5:**
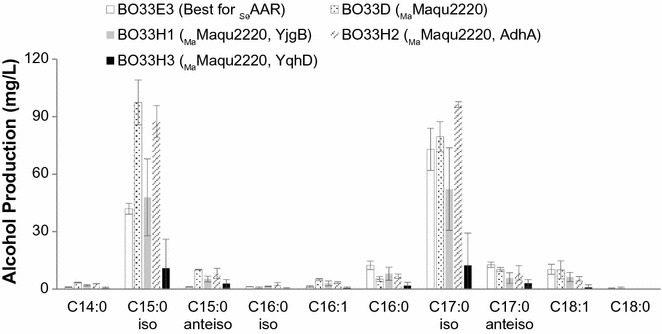
BLFL profiles of engineered *E. coli* strains containing Maqu2220. While strain BO33D does not contain additional *adh*, BO33H1-3 overexpressed *L. lactis adhA*, *E. coli yqhD*, and *E. coli yjgB*, respectively. BLFL profiles are compared with that of strains BO33E3 (the best performing strain in “Engineering alcohol formation modules in BCFA-producing strains” section). All cultures were supplemented with 1 g/L 4-methyl-2-oxopentanoic acid

During the course of this work, Haushalter et al. published the production of straight LCFLs in high titers by overexpression of a marine acyl-ACP reductase, Maqu2220 from *M. aquaeolei* VT8 [48]. Maqu2220 was first characterized as a fatty aldehyde reductase, reducing fatty aldehydes to fatty alcohols [49]. However, it was later confirmed to have acyl-ACP reductase activity and is capable of converting acyl-ACPs or acyl-CoAs directly to fatty alcohols [50], thus significantly simplifying the fatty alcohol pathway. We tested the efficiency of BLFL production via Maqu2220 by replacing the *S. elongatus aar* in strain BO33A with *maqu2220*, resulting in strain BO33D. When supplemented with 1 g/L of 4-methyl-2-oxopentanoic acid, BO33D produced 198 mg/L (89%) BC alcohols, 3.7-fold higher than that of strain BO33A (Fig. 5). Even compared with the previous best-performing strain BO33E3, the BLFL titer of strain BO33D enhanced by 1.5-fold. Most BLFLs (> 99%) were identified in the cell pellet, consistent with previous study [51]. Moreover, overexpression of the ADHs *yjgB*, *yqhD*, or *adhA* did not enhance BLFL titer (Fig. 5), further confirming the previous hypothesis that Maqu2220 contains a catalytic domain with ADH activity.

**Fig. 6:**
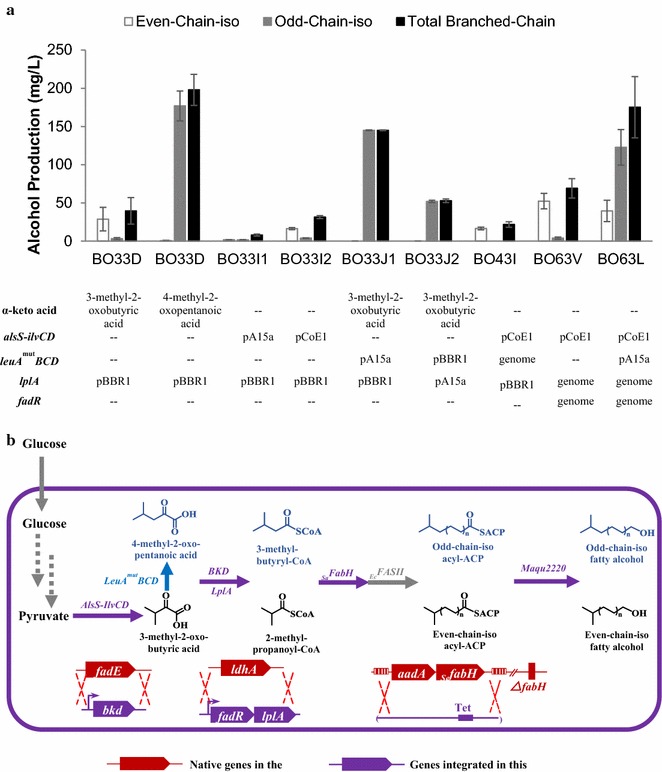
Optimizing α-keto acid biosynthetic pathways in alcohol-producing strains containing _Ma_Maqu2220. **a** Plasmids with different copy numbers and promoters were employed to bear the involved genes in different strains. The titers of even-chain-iso, odd-chain-iso, and total alcohol from all the strains containing α-keto acid biosynthetic pathways are compared with that of strain BO33D with either 1 g/L 3-methyl-2-oxobutyric acid or 1 g/L 4-methyl-2-oxopentanoic acid supplementation. **b** The complete pathways and genome modifications involved in strain BC63V (purple) and BC63L (purple and blue)

After confirming the capability of Maqu2220 to convert branched-chain acyl-ACPs to BLFLs, we sought to use Maqu2220 to produce BLFLs from glucose by applying the knowledge learned from optimizing the α-keto acid synthesis module. After constructing and validating strains, we found that the optimal expression level of each module mimicked those characterized using the *aar* pathway. First, the high-copy number-expression (pColE1) of the *alsS*-*ilvCD* operon increased even-chain-iso fatty alcohol production by 9.2-fold over the low-copy number-expression (pA15a) (as seen by comparing strains BO33I1 with BO33I2 in Fig. 6a). Second, when supplemented with 3-methyl-2-oxobutyric acid, the low-copy number-expression of the *leuA* ^*mut*^ *BCD* operon produced 2.8-fold more odd-chain-iso fatty alcohols than the medium-copy number-expression (as seen for strains OB33J1 and OB33J2 in Fig. 6a).

Next, we attempted to combine all three modules to produce BLFLs from glucose, which requires overexpression of 14 genes from 6 synthetic operons: P_tet_-*alsS*-*ilvCD*, P_BAD_-leu*A* ^mut^ *BCD*, P_lacUV5_-_*bs*_ *lpdV*-_*bs*_ *bkdAA*-_*bs*_ *bkdAB*-_*bs*_ *bkdB*, P_lacUV5_-*lplA*, P_ecfabH_-_Sa_ *fabH*, and P_lacUV5_-*maqu2220*. To reduce the burden imposed by multiple plasmids while maintaining a sufficient enzyme expression level, we tried to integrate some operons into the genome. Based on the findings above, we first created strain BC43 with a single copy *leuA* ^mut^ *BCD* from strain BC33. Using the Type II CRISPR-Cas9 system, we replaced the native promoters of the genomic *leuABCD* operon with the strong IPTG-inducible P_lacUV5_ promoter, and replaced the RBS of *leuA* (including all regulatory sites) with the previously-used strong synthetic RBS. Simultaneously, we replaced the *leuA*-coding gene with the feedback-resistant *leuA* ^mutant^. However, when the resulting strain BC43 was transformed with corresponding plasmids for fermentation, even-chain-iso products predominated over odd-chain-iso products (as seen for strains BO43I in Fig. 6a). This result indicated that the *leuA* ^mut^ *BCD* requires very fine-tuning and that a single copy of *leuA* ^mut^ *BCD* was insufficient to convert all the intracellular 3-methyl-2-oxobutyric acid to 4-methyl-2-oxopentanoic acid, further proving that pA15a::P_BAD_-leu*A* ^mut^ *BCD* provides optimal expression level for the α-keto acid synthesis module. Consistency in expression parameters indicates that the optimal levels derived in this work may be applicable in other systems requiring the use of an α-keto acid pathway.

Then, we chose *lplA* as the gene to be integrated into the host cell’s genome; low expression was previously determined to be sufficient to fully functionalize the BKD E2 subunit [27]. Furthermore, it has been demonstrated that even low-level overexpression of the global regulator of fatty acid metabolism *fadR* increases fatty acid production [52, 53]. Thus, *fadR* was cloned to the same operon of *lplA* under the control of the P_LacUV5_ promoter. The synthetic P_LacUV5_-*fadR*-*lplA* operon was then integrated into the native *ldhA* (involved in lactate production) site of the strain BC43, resulting in strain BC63 (Fig. 6b). Deletion of *ldhA* was expected to reduce lactate formation during fermentation [54].

Strain BC63 was then engineered to produce BLFL with controlled chain structure. To produce even-chain-iso BLFLs from glucose, we created strain BO63V by co-transforming the plasmids pB5k-*maqu2220* and pE2s-*alsS*-*ilvCD* into strain BC63 (Table 2, Fig. 6b). When fermented in the absence of α-keto acid, BO63V produced 52.4 mg/L of even-chain-iso fatty alcohols (Fig. 6a; Additional file 1: Figure S4). To produce odd-chain-iso fatty alcohols from glucose, the plasmid pA8c-*leuA* ^mut^ *BCD* was further transformed to strain BO63 V (Fig. 6b). The resulting strain BO63L produced 122 mg/L of odd-chain-iso BLFLs, comprising 70% of the total BLFLs (Fig. 6a; Additional file 1: Figure S4). Although the odd-chain-iso BLFL pathway is less carbon efficient than the even-chain-iso BLFL pathway due to the loss of a CO_2_ during 4-methyl-2-oxopentanoic acid biosynthesis, the _Sa_FabH has higher specificity towards 3-methylbutyryl-CoA (the precursor for odd-chain-iso products) than 2-methyl-propanoyl-CoA (the precursor for even-chain-iso products) [28], thus leading to higher odd-chain-iso BLFL titer.

**Fig. 7:**
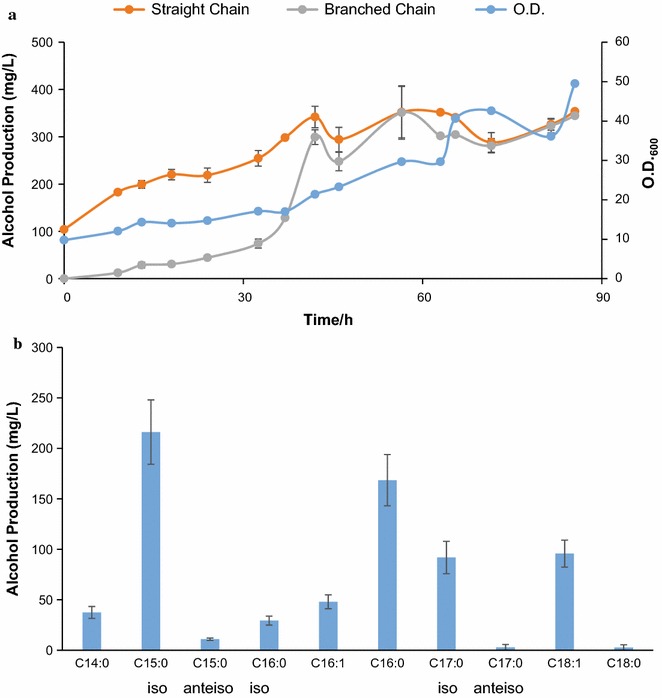
Fed-batch production by strain BO63L. **a** Time-course plots of straight LCFL and BLFL titer and cell density. **b** BLFL profile at 85 h during fed-batch fermentation

Finally, we assessed the performance of strain BO63L in a 1 L glucose fed-batch fermenter (“Fermentation” section). After 85 h of cultivation, BLFLs accumulated to 350 mg/L (Fig. 7a). The concentration of 14-methyl-pentadecanol, the most abundant product, reached 217 mg/L (Fig. 7b). Interestingly, straight LCFLs accumulated rapidly after induction, while BLFL titers did not increase until 32 h (Fig. 7a). We suspect that the initial straight-chain alcohol accumulation may result from the leaky expression of Maqu2220 prior to induction. Further optimization of fermentation conditions (such as pH, air flowing rate, stirring rate, etc.) might improve the percentage and yield of BLFL. Overall, these results demonstrate the potential of strain BO63L for high-titer production of BLFLs and suggest that further studies might lead to its use on an industrial scale.

## Discussion

Although alcohol-producing pathways have been previously engineered to produce straight LCFLs and short-chain alcohols, little effort has been made to produce BLFLs. In this work, we characterized the capability and substrate specificity of four different alcohol-producing pathways for BLFL production. We demonstrated the highly selective production of two types of BLFLs (odd-chain-iso and even-chain-iso) by engineering the upstream pathways for precursor synthesis. We obtained high BLFL proportions out of total fatty alcohols (strain BO63L, yielded 80%). We also obtained comparable BLFL titers (strain BO63L, 175 mg/L in the absence of any precursor) to BCFA titers, despite the extensive additional engineering required for BLFLs production. The modular engineering strategy allowed us to apply the knowledge learned from the AAR pathway to the new identified Maqu2220 pathway for rapid optimization, quickly yielded strain BO63L that produced 350 mg/L BLFLs in a fed-batch fermenter. Because BLFLs can be directly used in skin-care or sunscreen products, and are good candidates for diesel fuels, our ability to produce specific BLFL species in high percentages may directly benefit the cosmetics and bioenergy fields.

Meanwhile, we partitioned the complete pathway into three modules: a precursor formation module (the α-keto acid synthesis module), an acyl-CoA activation and malonyl-ACP consumption module (the acyl-ACP generation module), and a final product synthesis module (the alcohol formation module). Each module can be separately engineered and tuned for novel chemical synthesis. Furthermore, when the tuned modules are combined, the expression level of each module can be tuned to avoid imbalance and to improve product titer. We demonstrated consistent expression parameters that result in optimal productivities, which can be used for the production of other chemicals. Together with previous successes in modular engineering of the isoprenoid pathway [55], the FFA pathways [56] and the ester pathways [57], we confirm that modular pathway engineering is an effective approach to improve product titer and yield.

The top-performing strain BO63L produced 350 mg/L BLFLs in fed-batch fermenter, still low compared to that of the best straight LCFL-producing strain (3.82 g/L in shake flask) [48]. Our current pathway is not limited by α-keto acid because increasing the supplemented 4-methyl-2-oxopentanoic acid from 1 to 8 g/L did not increase BLFL titer (Additional file 1: Figure S3). Furthermore, conversion of acyl-ACPs to BLFLs is not likely to be the limiting step due to the high conversion efficiency of Maqu2220 [48]. Thus, we believe the current bottleneck lies in the acyl-ACP generation module, which requires further engineering to improve BLFL titer and yield. Previous engineering strategies developed to optimize free fatty acids production could be potentially used to further improve BLFL yield. These strategies include modulation of the fatty acid and the phospholipid biosynthesis pathway [58], implementing synthetic control systems to dynamically regulate pathway gene expression [52, 59–61], and enriching the high-performing subpopulation using PopQC [60].

## Conclusions

We have constructed and tested the capability of four different alcohol-producing pathways for BLFL production in engineered *E. coli*. Moreover, by engineering the α-keto acid biosynthetic pathways and balancing the expression levels of three different modules, we achieved BLFL titers of up to 350 mg/L from glucose, and BLFL percentages up to 79%. Overall, this work generates pathways and knowledge for the production of BLFLs in high percentages that will have broader industrial applications than straight LCFLs.

## Additional file

**Additional file 1: Figure S1.** Fatty alcohol profiles of strains involved in Fig. 3b. **Figure S2.** Fatty alcohol profiles of strains involved in Fig. 4b. **Figure S3.** Odd-chain-iso fatty alcohol production in strain BO33D with different concentrations of 4-methyl-2-oxopentanoic acid supplementation. The data has been normalized to the odd-chain-iso fatty alcohol production in strain BO33D with 1 g/L 4-methyl-2-oxopentanoic acid supplementation. **Figure S4.** Fatty alcohol profiles of strains involved in Fig. 6a.

## Abbreviations

BLFL: branched-long-chain fatty alcohol
ACP: acyl carrier protein
AAR: acyl-ACP reductase
CAR: carboxylic acid reductase
ACR: acyl-CoA reductase
FFA: free fatty acid
ADH: alcohol dehydrogenase
LCFL: long-chain fatty alcohol
BCFAs: branched-chain fatty acids
BKD: branched-chain α-keto acid dehydrogenase complex
FabH: β-ketoacyl-acyl-carrier protein
IPTG: β-d-1-thiogalactopyranoside
aTc: anhydrotetracycline
BSTFA: *N*,*O*-bis(trimethylsilyl)trifluoroacetamide
