# Supplementary material for: Modular pathway engineering for the microbial production of branched-chain fatty alcohols
Source: Biotechnol Biofuels. 2017 Oct 27;10:244. doi: 10.1186/s13068-017-0936-4 (PMC5658922; doi:10.1186/s13068-017-0936-4)
Supplement: Supplementary file 1 — Additional file 1: Figure S1. Fatty alcohol profiles of strains involved in Fig. 3b. Figure S2. Fatty alcohol profiles of strains involved in Fig. 4b. Figure S3. Odd-chain-iso fatty alcohol production in strain BO33D with different concentrations of 4-methyl-2-oxopentanoic acid supplementation. The data has been normalized to the odd-chain-iso fatty alcohol production in strain BO33D with 1 g/L 4-methyl-2-oxopentanoic acid supplementation. Figure S4. Fatty alcohol profiles of strains involved in Fig. 6a. [file 13068_2017_936_MOESM1_ESM.docx]

**Additional Materials**

**Modular pathway engineering for the microbial production of branched-chain fatty alcohols**

**Authors:** Wen Jiang^a^, James B. Qiao^a^, Gayle J. Bentley^b,d^, Di Liu^a^, Fuzhong Zhang^a-c*^

^a^Department of Energy, Environmental and Chemical Engineering

^b^Division of Biological & Biomedical Sciences

^c^Institute of Materials Science & Engineering

Washington University in St. Louis, Saint Louis, MO 63130, USA

Current address: ^d^National Bioenergy Center

National Renewable Energy Laboratory, Golden, CO 80401, USA

**^*^ Correspondence:**

Dr. Fuzhong Zhang

1 Brookings Drive, Campus Box 1180, St. Louis, MO, 63130, USA

[fzhang@seas.wustl.edu](mailto:fzhang@seas.wustl.edu)

Tel: 314-935-7671

**Figure S1. Fatty alcohol profiles of strains involved in Figure 3B.**

**Figure S2. Fatty alcohol profiles of strains involved in Figure 4B.**

**Figure S3. Odd-chain-iso fatty alcohol production in strain BO33D with different concentration of 4-methyl-2-oxopentanoic acid supplementation.** The data were normalized to the odd-chain-iso fatty alcohol production in strain BO33D with 1g/L 4-methyl-2-oxopentanoic acid supplementation.

**Figure S4. Fatty alcohol profiles of strains involved in Figure 6A.**
